# A Case of Complex Partial Seizures Presenting as Acute and Transient Psychotic Disorder

**DOI:** 10.1155/2019/1901254

**Published:** 2019-05-02

**Authors:** Sadia Sultan, Ebtihaj Omar Fallata

**Affiliations:** Department of Psychiatry, Institute of Mental Health, Hyderabad, India

## Abstract

**Introduction:**

Complex partial seizures are focal (CPS) (i.e., start in one area of the brain) and associated with impairment in consciousness. Most of them arise in the temporal region and are characterized by aura, impaired consciousness, and automatisms. CPS that arise in temporal region are most often misdiagnosed as primary psychiatric illness.

**Case Report:**

A 25-year-old male presented with fluctuations in consciousness, aggressive behaviour, hallucination, and delusions of grandeur lasting a few hours. He was diagnosed with acute and transient psychotic disorder according to ICD10 criteria and was treated with intramuscular haloperidol 10mg BID followed by oral olanzapine 10mg. Computed tomography of brain and electroencephalogram were normal. After 15 days he presented again to the outpatient department with complaints of aggressive behaviour and sensory misinterpretations. Video electroencephalogram was recommended, which was not done due to financial constraints. The diagnosis was reconsidered and he was started on oral carbamazepine due to high clinical suspicion, of complex partial seizures, in spite of lack of EEG evidence. He responded well to antiepileptic and symptom remission has maintained well.

**Conclusion:**

Patients presenting with psychosis need careful diagnostic evaluation for other possibilities.

## 1. Introduction

The intermingling of psychiatric symptoms and epilepsy goes back to the time when the term ‘lunatics' was used for people suffering from epilepsy [1]. Focal seizures that start in one hemisphere of brain and are associated with impaired consciousness are referred to as complex partial seizures (CPS) also called temporal lobe seizures (TLE) or psychomotor seizures. CPS is characterized by aura, impaired consciousness, and automatisms [2]. Among all epilepsies, CPS of temporal origin most often present with psychopathology [1, 2]. The incidence rate of epilepsy ranges from 34.7 to 54.3 per 100,000 population. TLE accounts for 10.4 per 100,000 population and is considered the most common type of partial epilepsy [3]. The overall prevalence of psychiatric disturbances in epileptic patients can be estimated between 20 and 30% per cent [4] Often due to affective, behavioural, and cognitive symptoms CPS is misdiagnosed as primary psychiatric disorder. Usually these symptoms present with others, which are atypical for the diagnosis of primary psychiatric illness, such as macropsia, micropsia, gustatory and olfactory hallucinations, intense short-lived delusions, and dejavu phenomenon [5]. CPS presents as aura followed by ictal, postictal, and interictal states. Aura presents as epigastric churning, dejavu phenomenon, recurrent intrusive thoughts, hallucination of unpleasant smell, fear, and tachycardia. This is followed by automatisms (semipurpose seemingly automatic movements) of mouth and hands observed in ictal state. Examples include lip smacking, chewing, and swallowing, picking at buttons, and repetitive hand movements. Patients usually appear semireactive, in a trance-like state, and have no recollection of automatisms. Nevertheless, in some patients walking, running, and nondirected violent behaviour may occur as a part of automatisms [5, 6]. Fugue or twilight state refers to an altered state of consciousness with motoric activity followed by amnesia for the event and personal identity. This state may last from hours to days and is encountered in complex partial seizures nonconvulsive status epilepticus (CPNSE), sometimes as sole manifestation [7]. CPNSE is also called ictal psychosis due to its clinical presentation which is characterized by visual and auditory hallucinations, delusions, illusions, ideas of reference, paranoia, and behaviour that is confused, perplexed, and agitated which may last from hours to months [8]. Complex partial status epilepticus (CPSE) represented approximately 5% of the total number of convulsive and nonconvulsive episodes and 35% of nonconvulsive episodes [9]. Other studies have demonstrated that CPSE accounts for 10–40% of all cases of nonconvulsive status epilepticus which accounts for 20% of all status epilepticus [10]. Postictal state may present as postictal confusion or dysphasia. Postictal psychosis may follow the seizure and accounts for 25% of all psychosis associated with epilepsy and occur after a lucid interval of 1 to 6 days [11].  Table 1 is depicting the differences between postictal psychosis and CPNSE.

## 2. Case Report

A 25-year-old male, labourer, inhabitant of Hyderabad, India, presented with history of abnormal behaviour, delusions, hallucinations, and aggression beginning a few hours prior to presentation. The onset was acute and progressive in nature. According to his wife, he suddenly stood up and started picking at his clothes and searching for something in a drawer; at the same time he was not responding to her calls. He was also reported to be sniffing like a dog. When she intervened, he became aggressive and physically assaulted her. He was confused and did not seem to respond to the surroundings. No history of fever, fall, head trauma, or substance abuse was reported. On examination, patient's vitals, and all systems including neurological system were normal. Rapport could not be established as the patient appeared confused and perplexed and was not responding to any questions. He appeared to be agitated and hallucinating as he was talking to self. He was admitted and on further evaluation it was revealed that although his speech was sparse, on many occasions he said God was talking to him and he could also see Him; hence he has the power to do anything. He only intermittently recognized family members. His orientation to time, person, and place was lost on many occasions. However, there were bouts of aggressive behaviour which was nondirected and seemed purposeless. All routine investigations were within normal limits including electrolytes, computed tomography (CT) brain, and electroencephalogram (EEG). The patient was diagnosed as acute transient psychotic disorder according to the tenth revision of the International Classification of Mental and Behavioural Disorders (ICD 10) criteria. He was given intramuscular, haloperidol 10mg IM twice for 2 days. Improvement was noticed in terms of sleep, control of aggression, and orientation. Surprisingly, all the psychotic symptom improved within 2 days. Later he was switched to oral olanzapine 10mg OD due to mild extrapyramidal side effects and was discharged after 3days. He returned after 15 days with complaints of abnormal behaviour. According to his wife he was aggressive, entered a restaurant, and broke the furniture. Patient had no memory of aggressive behaviour. On history taking it was revealed that the present episode lasted only for few minutes. The nature of symptoms as described by patient is feeling of twisting in the abdomen and felt that the bubbles of rain had become very large since it was raining that day. At the same time, he had a thought of infidelity about his wife and did not remember the subsequent episode of aggression which was reported by the wife. On examination the vitals were normal and mental status examination (MSE) seemed to be absolutely normal. Moreover, on further enquiry the wife recollects several such episodes of short-lived abnormal behaviour for the last two years. Video EEG monitoring was recommended in the higher level medical care facility, which he could not do due to financial constraints. He was started on oral carbamazepine 200mg BID on clinical suspicion of CPS, which was later increased gradually to 1000mg per day. Simultaneously olanzapine was tapered off. The patient has been maintained on 1000mg oral carbamazepine per day for the last 2 years with no relapse in the symptoms.

## 3. Discussion

Feeling of churning in the abdomen, macropsia, olfactory hallucinations, and recurrent intrusive thoughts can be seen as a part of aura in temporal lobe epilepsy. The twisting feeling in abdomen as reported by the patient in the present case can be considered as autonomic aura. The phenomenon of bubbles becoming large could be considered macropsia which is a sensory misinterpretation; the thought of infidelity is more likely to be repeated intrusive thought and sniffing is an olfactory hallucination. These symptoms are all a part of aura as seen in the present case. Activation of reciprocal connections between mesial and neocortical temporal cortex produce aura; epigastric aura is more often encountered in seizures of right temporal foci [13]. Picking at clothes, searching, and aggressive behaviour are automatisms which are of mesial temporal lobe origin and bitemporal spread heralds the alteration in consciousness [13]. Most of the time the aura is followed by abnormal aggressive behaviour which is nondirected violence which may be described as automatisms. CPNSE of temporal origin may present with symptoms remarkably similar to primary psychiatric illness [14]. The abnormal behaviour lasting for 2 days mimicking a psychotic episode with fluctuations in consciousness, sparse speech, drowsiness and bouts of aggression, delusions, and hallucinations can be considered as a CPNSE of temporal origin in the present case.

Atypical CPNSE attacks are characterized by fluctuating restless agitated behaviour with delusions and hallucinations mostly with religious content which may last for hours to days or even months [14] as seen in the present case. CPNSE shows prolonged epileptic discharge on intracranial stereoelectroencephalography (SEEG) in hippocampus and mesial temporal structures sometimes without changes in scalp EEG [14]. Similarly, our patient did not show any changes in scalp EEG during the psychotic behaviour. Although video EEG or SEEG was not done to confirm the possibility of CPNSE due to lack of availability at our centre, the diagnosis is considered only based on clinical suspicion. The psychotic behaviour encountered in the present case cannot be considered as postictal psychosis as the lucid interval is not reported. Although we could not confirm the diagnosis of CPS, we would still like to consider the tentative diagnosis of CPS of temporal origin depending on the episodic nature of the abnormal behaviour, clinical suspicion, and improvement with antiepileptic medication. All antipsychotics are known to reduce the seizure threshold and increase the propensity for the seizure. This tendency varies between the different antipsychotics and appears to be dose dependent. Among the typical antipsychotics, haloperidol has the least propensity to precipitate a seizure. However, among the atypical antipsychotics, clozapine is the most epileptogenic with seizures being reported in 0.3% to 5% of people treated with therapeutic doses. Similarly, olanzapine has also showed occurrence of seizure in 0.24% of patients without epilepsy [15]. Incidence of seizure is more likely if the dose of olanzapine exceeds 20mg, especially in individuals with epilepsy, according to Degner et al. [16]. However, in that case series, it was also reported that recurrence of seizures was minimally reduced or unchanged during olanzapine treatment, while participants in the haloperidol group had increased seizures compared to the baseline. Nevertheless, limited evidence in the form of one RCT and a few descriptive studies have shown improvement of psychotic symptoms of epilepsy with antipsychotics [17]. In the present case it may be argued that the symptom improvement was due to spontaneous remission and, in fact, the use of haloperidol might have delayed the spontaneous remission. The CPNSE in this case lasted for 2 to 3 days, which otherwise might have remitted within hours if haloperidol was not used. This highlights the importance of video EEG in department of psychiatry for diagnostic clarity. Furthermore, the use of olanzapine might have played a role in lowering the seizure threshold and triggering another episode of seizure within 15 days although the dose was not exceeding 20 mg.

In conclusion this case highlights the importance of history taking and clinical evaluation of psychotic patients for causes other than primary psychiatric illness and the need for video EEG monitoring in the psychiatric department. It also highlights the importance of consideration of diagnosis of CPS even when the EEG is normal based on clinical suspicion and improvement with antiepileptic medication.

## Figures and Tables

**Table 1:** Shows contrasting features of postictal psychosis and CPNSE.

Postictal Psychosis	CPNSE
Lucid interval 12 hrs – 6 days [11]	No lucid interval [11]
Lasts for 12 hours – less than 2 months. [12]	Lasts for few hours to months. [12]
Mental state characterised by delusions and hallucinations in clear consciousness [12, 13]	Hallucinations, mood symptoms and delusions with impaired consciousness [12, 13]
Episodes of psychosis often without confusion and delirium, following a cluster of seizures [12]	Major features are confusion, delirium and aggression [12]
No evidence of EEG changes [12]	Evidence of EEG changes [12]
